# Ultrasound-assessed abdominal fat distribution and its relation to sarcopenia parameters in community-dwelling young older adults: a cross-sectional study

**DOI:** 10.3389/fendo.2026.1888492

**Published:** 2026-06-19

**Authors:** Claudia Jiménez-ten Hoevel, Iolanda Gironès, Júlia Nicolás-Marzo, Maria Besora-Moreno, Judit Queral, Rosa M. Valls, Patricia Pérez-Matute, Maria-José Motilva, Elisabet Llauradó, Rosa Solà, Anna Pedret

**Affiliations:** 1 Functional Nutrition, Oxidation, and Cardiovascular Diseases Group (NFOC-Salut), Facultat de Medicina i Ciències de la Salut, Universitat Rovira i Virgili, Reus, Spain; 2 Institut de Recerca Biomèdica Catalunya Sud, Hospital Universitari Sant Joan de Reus, Reus, Spain; 3 Lifestyles, Microbiota and Health Group (GESMUD), Health Science Faculty, University of La Rioja, La Rioja, Spain; 4 Instituto de Ciencias de la Vid y del Vino-ICVV (Consejo Superior de Investigaciones Científicas-CSIC, Universidad de La Rioja, Gobierno de La Rioja), Finca La Grajera, Logroño, Spain; 5 Department of Intern Medicine, Hospital Universitari Sant Joan de Reus, Reus, Spain

**Keywords:** body composition, sarcopenia, subcutaneous adipose tissue (SAT), ultrasound, visceral adipose tissue (VAT)

## Abstract

**Background:**

Ageing is associated with significant changes in body composition, including increased abdominal adiposity and reduced skeletal muscle strength, mass, and physical performance, known as sarcopenia. However, the association of specific abdominal fat distribution in relation to sarcopenia parameters remains poorly understood.

**Objective:**

To assess abdominal fat distribution by ultrasound (US) and explore its association with sarcopenia parameters in community-dwelling young older adults aged 60–74 years.

**Methods:**

This cross-sectional study (ClinicalTrials.gov: NCT06871384) included 72 participants (68.1% women). Abdominal fat distribution was assessed by US, measuring total abdominal fat, visceral adipose tissue (VAT), subcutaneous adipose tissue (SAT), and VAT/SAT ratio. Muscle strength was evaluated using a hand grip dynamometer, muscle mass indices [skeletal muscle mass Index (SMI) and appendicular skeletal muscle mass index (ASMI)] by BIA and physical performance by gait speed (GS). Linear regression analysis was adjusted by sex, age and physical activity.

**Results:**

Participants were 67.11 ± 4.4 years old [68.1% (49/72) women], 86.1% (62/72) were non-sarcopenic, and 71.4% (50/72) were non-obese. After regression analysis, in all population, VAT was positively associated with muscle mass indices. SAT was negatively associated with GS and relative handgrip strength (HGS/BW). In men, VAT was positively associated with ASMI and inversely associated with HGS/BW. In women, SAT was positively associated with ASMI and inversely associated with HGS/BW and GS, whereas VAT was positively associated with SMI and ASMI and inversely associated with HGS/BW.

**Conclusion:**

US-assessed abdominal fat distribution showed sex-specific associations with sarcopenia parameters. In men and in women, VAT was positively associated with muscle mass and negatively associated with muscle strength. Whereas in women, SAT was the only fat depot negatively associated with physical performance. These results suggest that abdominal fat distribution may influence physical performance and relative muscle strength in ageing populations, highlighting the importance of evaluating abdominal fat distribution when assessing sarcopenia parameters.

## 1 Introduction

Ageing is accompanied by profound and clinically relevant changes in body composition, including increased adipose tissue accumulation and the progressive decline of muscle mass (1). The progressive decline of muscle mass is considered a major determinant of frailty, functional impairment, and adverse health outcomes in older adults (2). According to the European Working Group on Sarcopenia in Older People (EWGSOP) sarcopenia is defined based on three key parameters: skeletal muscle strength, muscle mass and physical performance (2, 3). The EWGSOP1 (2010) consensus defined presarcopenia as presenting low muscle mass, confirmed sarcopenia when low muscle mass and low muscle strength or low physical performance was present, and sarcopenia severe when all three parameters were low (3). The updated EWGSOP2 (2019) consensus redefined the classification of sarcopenia, classifying probable sarcopenia when low muscle strength is present, confirmed sarcopenia when low muscle strength and low muscle mass or muscle quality is present, and sarcopenia severe when all parameters (muscle strength, muscle mass or quality, and physical performance) are low (2).

Regarding adipose tissue, traditional assessments such as body mass index (BMI) and waist circumference (WC), often fail to distinguish between distinct fat depots (4). However, the regional distribution of adipose tissue plays a key role in metabolic dysfunction and age-related muscle decline (5).

Abdominal adipose tissue comprises two major compartments: subcutaneous adipose tissue (SAT) and visceral adipose tissue (VAT) (6). VAT is a heterogeneous depot that includes several anatomically and functionally distinct compartments, such as preperitoneal, omental, mesenteric, and perirenal fat, each characterized by specific vascularization, metabolic activity, and endocrine profiles (7, 8). SAT, formerly regarded as a homogeneous compartment, is recognized into superficial and deep SAT layers, anatomically partitioned by Scarpa’s fascia, with different metabolic effects. Superficial SAT layer is linked to healthier metabolic profiles, while deep SAT layer is associated with liver fat accumulation and poorer metabolic health (9).

In terms of distribution, SAT represents approximately 80% of total body adipose tissue, whereas VAT accounts for a smaller proportion, estimated at 10–20% in men and 5–10% in women (6). Importantly, aging is associated with a redistribution of abdominal adipose tissue, characterized not only by an overall increase in fat mass but also by a preferential accumulation of abdominal fat, including both VAT and SAT, with a more pronounced expansion of SAT in women (10).

In large population−based studies, indices of visceral adiposity, such as the weight-adjusted-waist index (WWI) and Visceral Adiposity Index, are positively associated with sarcopenia prevalence, independent of total adiposity and other confounders, such as lifestyle factors (e.g., smoking, alcohol consumption, and physical activity), socioeconomic status, and cardiometabolic comorbidities such as diabetes and hypertension (11, 12). Moreover, when sarcopenia and obesity coexist, forming a phenotype termed sarcopenic obesity, which exacerbates morbidity and mortality beyond the effects of either condition alone (13). Emerging epidemiological evidence supports a strong positively association between VAT accumulation and sarcopenia risk (11, 14). Increased accumulation of visceral fat depots may contribute to muscle loss through chronic low-grade inflammation and insulin resistance (15). In contrast, the role of SAT depot on sarcopenia remains less clear and comparatively underexplored. While some evidence suggests that SAT may exert protective or neutral metabolic effects (8), its specific contribution to muscle mass decline and functional impairment during aging has not been fully elucidated.

Beyond VAT and SAT, ectopic fat infiltration within and between muscle fibres (inter− and intramuscular adipose tissue) has been shown to compromise muscle quality, contractile efficiency, mitochondrial function, and contributes to insulin resistance, all of which are linked to diminished strength and physical performance, independent of muscle mass (15, 16). VAT is to a greater extent than SAT strongly associated with ectopic fat infiltration in skeletal muscle due to its pro-inflammatory and metabolically active profile that promotes lipid overflow and muscle insulin resistance (17).

Accurate quantification of abdominal adiposity is therefore critical for elucidating these associations. Computed tomography (CT) and magnetic resonance imaging (MRI) are the gold standards for measuring VAT and SAT, but their cost, limited accessibility, and in the case of CT, ionizing radiation, restrict their use in clinical practice and large population studies (18). To provide more available alternative measures abdominal ultrasound has emerged as a feasible, non−invasive, cost−effective alternative for estimating abdominal fat depots, including VAT and SAT (4, 18).

Despite that different fat depots may exert distinct metabolic and mechanical influences on musculoskeletal function, the specific relationships between VAT and SAT, and individual sarcopenia parameters, including skeletal muscle strength, mass, and physical performance, remain unclear.

Given the age-related redistribution of abdominal adipose tissue, we hypothesize that alterations in both visceral and subcutaneous fat compartments are associated with sarcopenia-related parameters. In this context, ultrasound-based assessment of abdominal fat distribution may offer a practical and non-invasive tool for the early identification of sarcopenia parameters, particularly considering the potential underdiagnosis of sarcopenia in community-dwelling older adults.

Therefore, this study aims to investigate the association between abdominal adipose tissue distribution (VAT and SAT), assessed by ultrasound, and sarcopenia-related parameters in community-dwelling older adults, to clarify the link between fat depot-specific influence on muscle health.

## 2 Methods and procedures

### 2.1 Study design and participants

The present study is a cross-sectional study based on the baseline data of the *WinAging* Randomized Controlled Trial (RCT). Baseline assessments were performed between May 2025 and March 2026 in Reus (Spain), and visits held at Centre Mèdic Quirúrgic (CMQ). The WinAging Study aims to evaluate the chronic effects of red wine polyphenols (mean dose of 150 mg/day), administered through a non-alcoholised red wine and in the context of a Mediterranean diet, in elderly and community-dwelling young old adults. The study followed the Helsinki Declaration and Good Clinical Practice Guidelines of the International Conference of Harmonization (ICH GCP), and was approved on 28/11/2024 by the Ethics Committee of the Pere Virgili Health Research Institute (IISPV) (256/2024) and registered on ClinicalTrials.gov (NCT06871384). All participants provided written informed consent.

### 2.2 Eligibility criteria

The inclusion criteria were as follows: a) men or women between 60–74 years old; b) sensory tolerance to red wine; c) prior to trial participation, signed informed consent.

The exclusion criteria were the following: a) men or women ± 75 years old; b) hypoglycaemia treatment or type 1 and type 2 diabetes mellitus diagnosed; c) anaemia (haemoglobin ≤13 g/dL in men and ≤12 g/dL in women); d) subjects diagnosed of intestinal disorders such as Chron’s disease, colitis ulcerous, and irritable bowel syndrome; e) to present a clinical active chronic disease; f) to present severe sarcopenia; g) to present cognitive impairment (MMSE ≤ 24 or clinical diagnosis of mild cognitive impairment or dementia); h) dietary allergies to Mediterranean foods (e.g., nuts), sulphites or nitrates; i) use of antioxidants supplements; j) regular consumers of red wine who do not agree to change the consumption of red wine with alcohol to non-alcoholised wine during the intervention; k) chronic alcoholism; l) current or past participation in a clinical trial or consumption of a research product in the 30 days prior to inclusion in the study; and m) failure to follow the study guidelines.

### 2.3 Assessment of abdominal fat distribution by ultrasound

**Figure 1:**
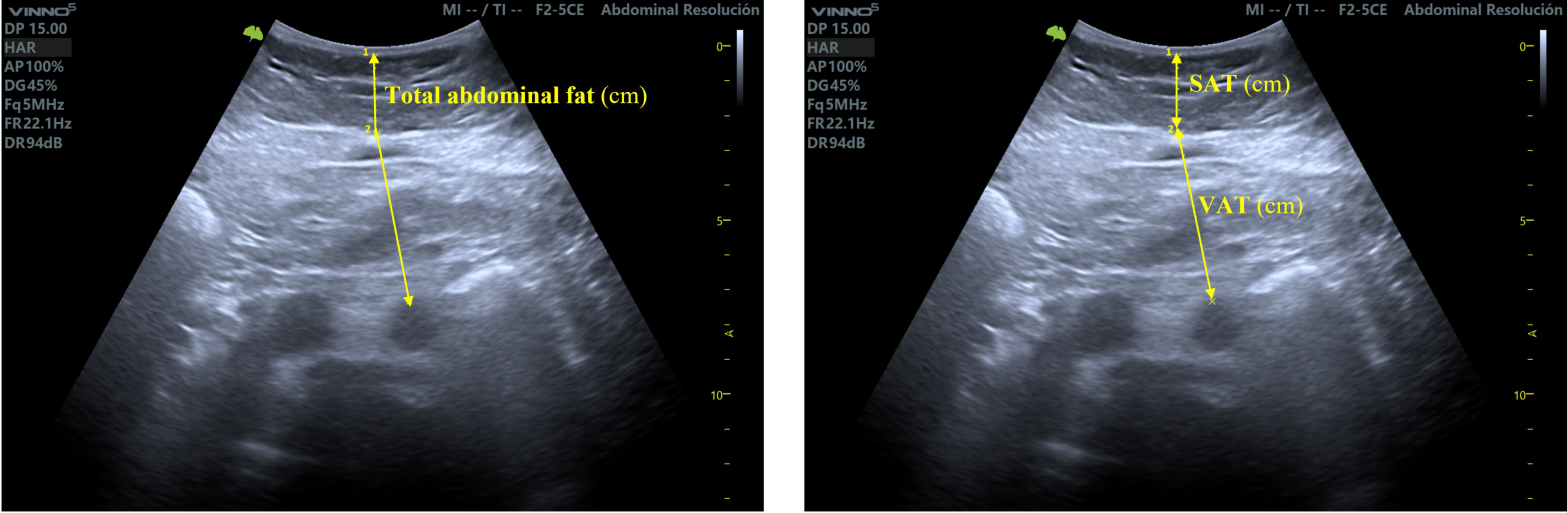
Ultrasound assessment of abdominal fat distribution. SAT, Subcutaneous Adipose Tissue; VAT, Visceral Adipose Tissue.

US measurements of SAT and VAT were taken in the supine position. The aortic bifurcation was first identified in the axial plane as the primary anatomical landmark. Once located, measurements were obtained at this level with minimal transducer pressure. As an initial reference, the probe was placed approximately 2 cm above the umbilical scar to facilitate localization of the L3–L4 region, where the aortic bifurcation is commonly found. Correct anatomical identification was confirmed by visualization of the iliac bifurcation in transverse view (18, 19). Scans were done during exhalation using a VINNO 5 device (Vinno (Suzhou) Co., Ltd., Suzhou, China) in the HAR-mode with the Abdominal Resolution preset at a frequency of 5 MHz with a convex transducer (F2-5CE). All ultrasound measures were carried out three times by a single researcher, and the mean was used for analysis. Total abdominal fat (cm), including SAT and VAT, was measured as the distance between the internal boundary of the dermis to the anterior wall of the aorta. SAT (cm), including superficial and profound SAT, was measured as the distance between the internal boundary of the dermis and the linea alba, and VAT (cm), including preperitoneal and intraperitoneal (omental and mesenteric) fat, but not perirenal fat, as the distance from the posterior face of the linea alba to the anterior wall of the aorta (18, 19) (**Figure 1**). VAT/SAT ratio was calculated.

### 2.4 Assessment of anthropometric parameters

Anthropometric parameters were obtained while the subjects were wearing lightweight clothing and no shoes. Trained dietitians measured the body weight (BW) and body composition of the subjects using a body composition analyser (Tanita MC 780-MA; Tanita Corp., Barcelona, Spain) and the height of the subjects using a wall-mounted stadiometer (Tanita Leicester Portable; Tanita Corp., Barcelona, Spain).

BMI was classified according to the classification for adults over 65 years old (20). BMI was classified as follows: 18.5–22 kg/m^2^ underweight; 22-26.9 kg/m^2^ healthy weight; 27-29.9 kg/m^2^ overweight; 30-34.8 kg/m^2^ obese class 1; 35-39.9 kg/m^2^ obese class 2; 40-40.9 kg/m^2^ obese class 3; and ≥50 kg/m^2^ obese class 4 (20).

The WC was measured at 2 cm above the umbilicus scar using a 150-cm anthropometric steel measuring tape (21). The waist (WC, cm) to height (cm) ratio (WHtR), the conicity index (CI), defined as WC (cm)/[0.109 x square root of body weight (kg)/height (m)], and the weight-adjusted-waist index (WWI), defined as WC (cm)/square root of body weight (kg), were calculated (11, 22, 23).

### 2.5 Assessment of sarcopenia parameters

Skeletal muscle strength based on handgrip strength (HGS; kg) was measured using a validated hydraulic handheld dynamometer (JAMAR^®^ Plus+ Dynamometer; Performance Health Supply, Inc., Cedarburg) (2). It was measured twice in the dominant hand and the average of the two values was calculated. HGS adjusted by BW (HGS/BW) assessed the relative strength, making it particularly useful for identifying sarcopenia in individuals where absolute strength alone may be misleading, especially those with higher BW and it was calculated (24).

Muscle mass was assessed by bioimpedance analysis (BIA), a segmental multifrequency body composition analyser (TANITA MC-780MA; Tanita Corp., Tokyo, Japan). The muscle mass quantity parameters assessed were the skeletal muscle mass (SM; kg), skeletal muscle mass index (SMI; kg/m^2^), appendicular skeletal muscle mass (ASM; kg), appendicular skeletal muscle mass index (ASMI; kg/m^2^), and phase angle (°). Also, the muscle quality was determined by the phase angle (°) assessed by BIA (2, 25).

Also, physical performance was measured twice by 4-meter gait speed (GS) and the average of the two values was calculated (2).

The cut-off points to determine low skeletal muscle strength were: <30 kg in men and <20kg in women, based on EWGSOP1 consensus (3); and <27kg in men and <16kg in women based on EWGSOP2 consensus (2). The cut-off points to determine low muscle mass parameters were: <8.87 kg/m^2^ of SMI in men and <6.42 kg/m^2^ of SMI in women based on EWGSOP1 consensus (3); and <20 kg of ASM in men and <15 kg of ASM in women, or <7 kg/m^2^ of a ASMI in men and <5.5 kg/m^2^ of ASMI in women based on EWGSOP2 consensus (2). A GS of ≤0.8 meters/second (m/s) was considered low physical performance based on both EWGSOP consensus (2, 3). Both consensuses were taken into account in order to compare the precision of each consensus to identify sarcopenic participants.

To determine sarcopenic obesity, the diagnostic criterion of the ESPEN and EASO consensus statement were employed (13). Participants were screened for having obesity (BMI ≥ 30 kg/m2 or WC ≥ 88cm in women and ≥ 102 cm in men) and low muscle strength based on handgrip strength employing the previous described cut-off point of EWGSOP2 (2, 13). If participants presented obesity, either by BMI or WC, and low muscle strength they were categorized as probable sarcopenic obesity (26). To confirm sarcopenic obesity, the criteria are to present obesity per BMI or WC, low muscle strength and altered body composition assessed by BIA: increased fat mass (> 31% in men and > 43% in women) and reduced muscle mass, determined as SM adjusted by BW (≤ 27 in men and ≤ 16 in women) (13). Taking into account the mean age of the study population, the BMI classification for older adults was used, as described in 2.4 section.

### 2.6 Assessment of cardiovascular risk factors

Systolic and diastolic blood pressure (SBP and DBP) were measured twice after 2–5 min of patient respite, seated, with a 1-min interval in between, using an automatic sphygmomanometer (OMRON M6 Comfort, HEM-7360-E; Peroxfarma, Barcelona, Spain). The pulse pressure (PP) was determined by the difference between the SBP and DBP readings (27).

Capillary blood samples were obtained by finger-stick using a sterile single-use lancet according to standardized point-of-care sampling procedures (28). The puncture site (lateral surface of the fingertip) was disinfected with 70% ethanol and allowed to dry completely. The first drop of blood was removed to reduce contamination with interstitial fluid.

HbA1c and lipid profile (total cholesterol, high-density lipoprotein cholesterol, low-density lipoprotein cholesterol, and triglycerides) were determined with cobas® b 101 system (Roche Diagnostics, Mannheim, Germany). Capillary blood was collected directly from the fingertip and applied immediately onto the dedicated reagent disc (HbA1c and lipid profile), according to the manufacturer’s instructions.

Fasting blood glucose was determined with OneTouch Select®Plus (LifeScan Europe GmbH, Zug, Switzerland). Capillary blood was collected directly from the fingertip and applied immediately onto the reactive strips, according to the manufacturer’s instructions.

Haemoglobin was determined with HemoCue® Hb 801 analyser (HemoCue AB, Ängelholm, Sweden). Capillary blood was collected directly from the fingertip and applied immediately onto the microcuvettes, according to the manufacturer’s instructions.

### 2.7 Assessment of physical activity

Physical activity was as assessed by the International Physical Activity Questionnaire (IPAQ-E), which comprises questions related to levels of physical activity based on time spent (hours or minutes/day) sitting, walking (> 10min), and conducting moderate, and vigorous physical activity, based on the last seven days. Physical activity was categorized as low, medium, and high categories (29).

### 2.8 Statistical analysis

Categorical variables were presented as percentages (%). The Normality was tested by Kolmogorov-Smirnov test and visual inspection of histograms and Q–Q plots. Continuous variables were presented as mean ± standard deviation (SD) (normal distribution) or median and interquartile range (IQR) (non-normal distribution), separated by sex, and compared by t-test or Mann-Whitney U test, depending on the nature of the variable.

Spearman correlation coefficients (r) were calculated to determine significant correlations between abdominal fat distribution assessed by ultrasound (total abdominal fat, SAT, VAT and VAT/SAT ratio) or by routinely applicable anthropometric parameters (BMI, WC, WWI, WHtR, CI), and sarcopenia parameters (SMI, ASMI, HGS, HGS/BW, and GS), interpreted as: ≤0.20 none, 0.21–0.40 weak, 0.41–0.60 moderate, 0.61–0.80 high, and ≥0.81 very high (30). Spearman correlation analyses were performed as an exploratory step to assess relationships between abdominal fat distribution variables and sarcopenia-related parameters. Given the exploratory nature of these analyses, no formal correction for multiple comparisons (e.g., Bonferroni or Holm adjustment) was applied. Therefore, correlation results should be interpreted cautiously in terms of potential type I error inflation. Linear regression models were subsequently performed to further examine these associations, adjusting for sex, age, and physical activity, and were fitted to assess the association between abdominal fat distribution assessed by ultrasound or routinely applicable anthropometric parameters and sarcopenia parameters. These models were conducted for all statistically significant Spearman correlations and were intended to evaluate whether associations persisted after adjustment for potential confounders.

All statistical analyses were performed using SPSS IBM (Corp. Released 2025. IBM SPSS Statistics for Windows, Version 31.0.0.0, IBM Corp., Armonk, NY). A p-value of <0.05 was considered significant.

## 3 Results

### 3.1 Characteristics of the study participants

Baseline characteristics of the study participants are presented in **Table 1**.

**Table 1:** Baseline characteristics of the study participants.

	All population (n=72)	Women (n=49)	Men (n= 23)	*p-*value*
Age, *years*	67.11 ± 4.39	67.40 ± 4.15	66.5 ± 4.92	0.373
Anthropometric measures				
Weight, *kg*	69.70 (17.30)	66.30 (11.50)	83.15 (22.88)	**<0.001**
Height, *m*	1.60 (0.35)	1.56 (0.24)	1.72 (0.20)	**<0.001**
BMI, *kg/m^2^*	26.80 (5.30)	27.85 (7.20)	25.90 (4.80)	0.316
Fat mass, *%*	33.0 (32.50)	34.40 (23.00)	25.05 (25.90)	**<0.001**
Waist circumference, *cm*	96.00 (13.00)	94.00 (15.00)	101.50 (13.63)	**0.001**
Waist-to-height ratio	0.59 (0.08)	0.59 (0.10)	0.59 (0.07)	0.950
Conicity index	1.32 (0.11)	1.31 (0.12)	1.35 (0.10)	**0.025**
Sarcopenia parameters				
Handgrip strength, *kg*	23.05 (12.20)	20.10 (5.46)	36.38 (9.41)	**<0.001**
Skeletal muscle mass, *kg*	26.10 (7.70)	25.00 (3.60)	33.60 (5.35)	**<0.001**
Skeletal muscle mass index, *kg/m^2^*	10.30 (1.84)	9.81 (1.11)	11.68 (2.12)	**<0.001**
Appendicular muscle mass, *kg*	18.00 (5.80)	17.30 (2.50)	24.75 (5.43)	**<0.001**
Appendicular muscle mass index, *kg/m^2^*	7.14 (1.39)	6.80 (0.76)	8.48 (1.64)	**<0.001**
Phase angle, *°*	5.40 (0.80)	5.00 (0.80)	5.80 (0.78)	**<0.001**
Gait speed, *m/s*	1.05 ± 0.20	1.02 ± 0.20	1.11 ± 0.20	0.169
Ultrasound measurements				
Total abdominal fat, *cm*	7.16 (3.24)	6.60 (2.68)	8.14 (3.68)	**0.019**
SAT thickness, *cm*	1.90 (1.14)	2.32 (1.01)	1.54 (0.79)	**0.005**
VAT thickness, *cm*	5.27 ± 2.25	4.65 ± 1.64	6.59 ± 2.81	**0.001**
VAT/SAT ratio	2.46 (1.60)	2.23 (1.12)	3.56 (3.15)	**<0.001**
Cardiovascular parameters				
Total Cholesterol, *mg/dL*	201.00 (45.00)	214.00 (48.00)	192.00 (41.75)	**0.006**
HDL-c, *mg/dL*	62.00 (16.00)	63.00 (15.00)	51.00 (21.75)	**0.001**
LDL-c, *mg/dL*	120.00 (38.00)	126.00 (38.00)	113.00 (28.25)	0.174
Triglycerides, *mg/dL*	101.00 (80.00)	106.00 (80.00)	89.50 (77.75)	0.584
Glucose, *mg/dL*	102.00 (13.00)	99.00 (15.00)	106.50 (9.75)	0.073
HbA1c, *%*	5.40 (0.50)	5.40 (0.40)	5.40 (0.40)	0.513
Haemoglobin, *mg/dL*	13.60 (1.50)	13.30 (1.80)	14.65 (2.25)	**0.004**
Systolic blood pressure, *mmHg*	125.00 (24.00)	118.00 (24.00)	132.50 (16.25)	**0.002**
Diastolic blood pressure, *mmHg*	83.00 (12.00)	81.00 (12.00)	84.50 (12.00)	0.081
Heart rate, *ppm*	66.28 ± 9.76	68.83 ± 9.32	60.80 ± 8.51	**0.010**

BMI, body mass index; SAT, subcutaneous adipose tissue; VAT, visceral adipose tissue; HDL-c, high density lipoprotein cholesterol; LDL-c, low density lipoprotein cholesterol. Values are expressed as mean ± standard deviation (SD) for variables with normal distribution or median and interquartile range (IQR) for variables with non-normal distribution. * Sex differences by t-test or Mann-Whitney U test, depending on the nature of the variable, a p-value <0.05 is statistically significant. Significant results are expressed in bold.

A total of 72 participants were included, 31.9% were men (n=23/72), and 68.1% were women (n=49/72). The mean ± SD age of participants was 67.11 ± 4.39 years, and the median (IQR) BMI was 26.80 (5.30) kg/m2. There were significant differences between sexes for anthropometric measures, US measurements, sarcopenia parameters, and cardiovascular biomarkers.

Frequencies of CVD risk factors and sarcopenia classification of the participants are presented in **Table 2**. There were significant differences between sexes for BMI, WC and sarcopenia parameters according to EWGSOP1 and EWGSOP2 consensus.

**Table 2:** Frequencies of cardiovascular disease risk factors and sarcopenia classification of the study participants.

	Total (n=72)	Women (n=49)	Men (n=23)	*p-*value*
Body mass index classification				<0.001
Underweight, *% (n)*	6.9 (5)	6.1 (3)	8.7 (2)	
Healthy weight, *% (n)*	43.1 (31)	51.0 (25)	26.1 (6)	
Overweight, *% (n)*	26.4 (14)	20.4 (10)	39.1 (9)	
Obese, *% (n)*	23.6 (17)	22.4 (11)	26.1 (6)	
Class 1, *% (n)*	19.4 (14)	18.4 (9)	21.7 (5)	
Class 2, *% (n)*	4.2 (3)	4.1 (2)	4.3 (1)	
Class 3, *% (n)*	0 (0)	0 (0)	0 (0)	
Waist circumference classification ^a^ (n=70)				0.009
Low risk, *% (n)*	34.3 (24)	31.3 (15)	40.9 (9)	
High risk, *% (n)*	65.7 (46)	68.8 (33)	59.1 (13)	
**Sarcopenia parameters, EWGSOP1^b^**				
Low Handgrip Strength, *% (n)*	36.1 (26)	46.9 (23)	13.0 (3)	**0.018**
Low SMI, *% (n)*	0 (0)	0 (0)	0 (0)	
Low Gait speed, *% (n)*	11.3 (8)	14.3 (7)	4.5 (1)	**<0.001**
Sarcopenia classifications, EWGSOP1^b^				*0.059*
Non-sarcopenic, *% (n)*	63.9 (46)	53.1 (26)	87.0 (20)	
Presarcopenia, *% (n)*	36.1 (26)	46.9 (23)	13.0 (3)	
Sarcopenia, *% (n)*	0 (0)	0 (0)	0 (0)	
Severe Sarcopenia, *% (n)*	0 (0)	0 (0)	0 (0)	
Sarcopenia parameters, EWGSOP2^c^				<0.001
Low Handgrip Strength, *% (n)*	13.9 (10)	18.4 (9)	4.3 (1)	**<0.001**
Low AMM, *% (n)*	13.9 (10)	18.4 (9)	4.3 (1)	**<0.001**
Low ASMI, *% (n)*	1.4 (1)	0 (0)	4.3 (1)	**<0.001**
Low Gait speed, *% (n)*	11.3 (8)	14.3 (7)	4.5 (1)	**<0.001**
Sarcopenia classifications, EWGSOP2^c^				<0.001
Non-sarcopenic, *% (n)*	86.1 (62)	81.6 (40)	95.7 (20)	
Probable Sarcopenia, *% (n)*	11.1 (8)	14.3 (7)	4.3 (1)	
Sarcopenia, *% (n)*	2.8 (2)	4.1 (2)	0 (0)	
Severe Sarcopenia, *% (n)*	0 (0)	0 (0)	0 (0)	
Sarcopenic Obesity ^d^				
*BMI-defined Sarcopenic Obesity*				**<0.001**
Non-Sarcopenic Obesity	95.8 (69)	93.9 (46)	100 (0)	
Probable Sarcopenic Obesity	4.2 (3)	6.1 (3)	0 (0)	
Confirmed Sarcopenic Obesity	0 (0)	0 (0)	0 (0)	
*WC-defined Sarcopenic Obesity*				**<0.001**
Non-Sarcopenic Obesity	87.5 (63)	83.7 (41)	95.7 (22)	
Probable Sarcopenic Obesity	12.5 (9)	16.3 (8)	4.3 (1)	
Confirmed Sarcopenic Obesity	0 (0)	0 (0)	0 (0)	

SMI, Skeletal Muscle Index; AMM, Appendicular Skeletal Muscle Mass; ASMI, Appendicular Skeletal Muscle Index; EWGSOP, European Working Group on Sarcopenia in Older People; BMI, Body Mass Index; WC, Waist Circumference. ^a^ World Health Organization, 2008; ^b^ EWGSOP, 2010; Presarcopenia was determined by low handgrip strength, confirmed sarcopenia was determined by low handgrip strength and low muscle mass or low gait speed, and severe sarcopenia was determined by presenting low handgrip strength, muscle mass and gait speed; ^c^ EWGSOP2, 2019; Probable sarcopenia was determined by low handgrip strength, confirmed sarcopenia was determined by low handgrip strength and low muscle mass, and severe sarcopenia was determined by presenting low handgrip strength, muscle mass and gait speed ^d^ ESPEN and EASO Consensus Statement, 2022; Probable sarcopenic obesity was determined by presenting obesity, either by BMI or WC, and low handgrip strength, confirmed sarcopenic obesity was determined by presenting obesity, low muscle strength, high fat mass, and low skeletal muscle mass adjusted by body weight. * Gender differences by Chi-square test, a p-value <0.05 is statistically significant. Significant results are expressed in bold and borderline results are expressed in *italic*.

#### 3.1.1 Obesity classification

Regarding the BMI classification for adults aged ≥ 65 years old (20), 23.6% (n=17/72) of the participants presented obesity [22.4% women (n=11/49), and 26.1% men (n=6/23); p <0.001]. In contrast, 65.7% (n=46/72) of the study population presented elevated WC [68.8% women (n=33/49), and 59.1% men (n=13/23); p=0.009].

#### 3.1.2 Sarcopenia parameters

Regarding sarcopenia parameters, taking into account the EWGSOP1 consensus, 46.9% of women (n=23/49) and 13% of men (n=3/23), presented low HGS; p=0.018]. There are no cases found for low SMI.

When classified by EWGSOP1 consensus, 63.9% (n=46/72) were non-sarcopenic [53.1% (n=26/49) women and 87% men (n=20/23)], 36.1% (n=26/72) presented presarcopenia [46.9% women (n=23/49) and 13% men (n=3/23)], and nobody met the criteria for sarcopenia or severe sarcopenia.

The differences in sarcopenia classification according to EWGSOP1 were borderline significant when compared between sexes (p=0.059).

Regarding sarcopenia parameters, taking into account the EWGSOP2 consensus, 13.9% (n=10/72) presented low HGS [18.4% of women (n=9/49) and 4.3% of men (n=1/23); p<0.001], and 1.4% (n=1/72) presented low ASMI [0% of women (n=0/72) and 4.3% of men (n=1/23); p<0.001].

When classified by EWGSOP2 consensus, 86.1% (n=62/72) were non-sarcopenic [81.6% women (n=40/49) and 95.7% men (n=22/23)], 11.1% (n=8/72) presented probable sarcopenia [14.3% women (n=7/49) and 4.3% men (n=1/23)], and 2.8% (n=2/72) presented sarcopenia [4.1% women (n=2/49) and 0% men (n=0/23). There were no cases for severe sarcopenia.

The differences in sarcopenia classification according to EWGSOP2 were significant when compared between sexes (p<0.001).

Presence of low GS was of 11.3% (n=8/72) for all population, 14.3% for women (n=7/49), and 4.5% for men (n=1/23) (p<0.001).

#### 3.1.3 Sarcopenic obesity

Regarding sarcopenic obesity, there were no confirmed cases found. However, there were significant changes between the classification of probable sarcopenic obesity depending on the definition of obesity, if it is made by BMI or WC. According to the BMI classification, 4.2% (n=3/72) presented probable sarcopenic obesity [6.1% women (n=3/49) and 0% men (n=0/23); p<0.001], whereas when classified by WC, 12.5% (n=9/72) presented probable sarcopenic obesity [16.3% women (n=8/49) and 4.3% men (n=1/23); p<0.001].

### 3.2 Association between abdominal fat distribution assessed by ultrasound and sarcopenia parameters in all population

All Spearman correlation analysis results are described in **Supplementary File 1**.

Spearman correlation analysis in all population are presented in **Table 1** and linear regression analysis in all population are presented in **Table 3**.

**Table 3:** Linear regression between abdominal fat distribution and sarcopenia parameters in all population.

	Sarcopenia parameters									
	SMI, *kg/m^2^*		ASMI, *kg/m^2^*		HGS, *kg*		HGS/BW		GS, *m/s*	
	*β*	*p-value*	*β*	*p-value*	*β*	*p-value*	*β*	*p-value*	*β*	*p-value*
Abdominal fat distribution assessed by ultrasound										
Total abdominal fat, *cm*	0.379	**<0.001**	0.444	**<0.001**	-0.049	0.449	–	–	-0.329	**0.009**
Subcutaneous adipose tissue, *cm*	–	–	–	–	-0.008	0.901	-0.240	**0.013**	-0.300	**0.017**
Visceral adipose tissue, *cm*	0.368	**<0.001**	0.463	**<0.001**	-0.053	0.436	–	–	–	–
VAT/SAT ratio	0.174	0.106	0.303	**0.001**	-0.044	0.521	–	–	–	–
Abdominal fat distribution assessed by routinely applicable anthropometric parameters										
Body mass index, *kg/m^2^*	0.590	**<0.001**	0.628	**<0.001**	–	–	-0.478	**<0.001**	-0.380	**0.001**
Waist circumference, *cm*	0.524	**<0.001**	0.544	**<0.001**	0.006	0.927	–	–	-0.483	**<0.001**
Weight-adjusted-Waist Index	–	–	0.276	**0.003**	–	–	-0.304	**0.002**	-0.441	**<0.001**
Waist-to-height ratio	–	**-**	0.509	**<0.001**	–	–	-0.444	**<0.001**	-0.471	**<0.001**
Conicity Index	0.292	**0.004**	0.279	**0.002**	–	–	–	–	-0.441	**<0.001**

SMI, Skeletal Muscle Mass Index; ASMI, Appendicular Skeletal Muscle Mass Index, HGS, Handgrip Strength; BW, Body Weight; GS, Gait Speed; VAT, Visceral Adipose Tissue; SAT, Subcutaneous Adipose Tissue. Linear regression was adjusted for age, sex and total physical activity (METs/week), a p-value <0.05 is statistically significant. Significant results are expressed in **bold**, borderline results are expressed in *italic*.

After linear regression analysis, statistically significant positive associations were observed for total abdominal fat and SMI (*β* = 0.379, p <0.001), ASMI (*β* = 0.444, p <0.001) and an inverse association with GS (*β* = -0.329, p=0.009).

Regarding SAT, after linear regression, statistically significant inverse associations were observed for HGS/BW (*β* = -0.240, p=0.013) and GS (*β* = -0.300, p=0.017).

Regarding VAT, after linear regression, statistically significant positive associations were observed for SMI (*β* = 0.368, p <0.001) and ASMI (*β* = 0.463, p <0.001).

Lastly, regarding VAT/SAT ratio, after linear regression, statistically significant positive associations were observed for ASMI (*β* = 0.303, p=0.001).

### 3.3 Association between abdominal fat distribution assessed by ultrasound and sarcopenia parameters in men

Spearman correlation analysis in men are presented in **Table 2** and linear regression analysis in men are presented in **Table 4**.

**Table 4:** Linear regression between abdominal fat distribution and sarcopenia parameters in men.

	Sarcopenia parameters									
	SMI, *kg/m^2^*		ASMI, *kg/m^2^*		HGS, *kg*		HGS/BW		GS, *m/s*	
	*β*	*p-value*	*β*	*p-value*	*β*	*p-value*	*β*	*p-value*	*β*	*p-value*
Abdominal fat distribution assessed by ultrasound										
Total abdominal fat, *cm*	–	**-**	0.527	**0.019**	–	–	-0.531	**0.011**	-0.252	0.293
Subcutaneous adipose tissue, *cm*	–	–	–	–	–	**-**	–	–	–	**-**
Visceral adipose tissue, *cm*	–	–	0.525	**0.018**	–	**-**	-0.496	**0.018**	–	–
VAT/SAT ratio	–	–	0.457	**0.044**	–	**-**	–	–	–	–
Abdominal fat distribution assessed by routinely applicable anthropometric parameters										
Body mass index, *kg/m^2^*	0.846	**<0.001**	0.901	**<0.001**	–	–	-0.533	**0.009**	–	**-**
Waist circumference, *cm*	0.769	**<0.001**	0.825	**<0.001**	–	**-**	-0.634	**0.003**	–	**-**
Weight-adjusted-Waist Index	–	–	–	–	–	–	-0.459	0.129	–	–
Waist-to-height ratio	0.864	**<0.001**	0.851	**<0.001**	–	–	-0.557	**0.019**	–	–
Conicity Index	–	–	–	–	-0.076	0.781	-0.596	**0.022**	–	**-**

SMI, Skeletal Muscle Mass Index; ASMI, Appendicular Skeletal Muscle Mass Index, HGS, Handgrip Strength; BW, Body Weight; GS, Gait Speed; VAT, Visceral Adipose Tissue; SAT, Subcutaneous Adipose Tissue. Linear regression was adjusted for age and total physical activity (METs/week), a p-value <0.05 is statistically significant. Significant results are expressed in **bold**, borderline results are expressed in *italic*.

After linear regression, statistically significant positive associations were observed for total abdominal fat and ASMI (*β* = 0.527, p=0.019), and an inverse association with HGS/BW (*β* = -0.531, p=0.011).

Regarding VAT, after linear regression, statistically significant positive associations were observed for ASMI (*β* = 0.525, p=0.018), and an inverse association with HGS/BW (*β* = -0.496, p=0.018).

Lastly, regarding the VAT/SAT ratio, after linear regression, a statistically significant positive association was observed for ASMI (*β* = 0.457, p=0.044).

### 3.4 Association between abdominal fat distribution assessed by ultrasound and sarcopenia parameters in women

Spearman correlation analysis in women are presented in **Table 3** and linear regression analysis in women are presented in **Table 5**.

**Table 5:** Linear regression between abdominal fat distribution and sarcopenia parameters in women.

	Sarcopenia parameters									
	SMI, *kg/m^2^*		ASMI, *kg/m^2^*		HGS, *kg*		HGS/BW		GS, *m/s*	
	*β*	*p-value*	*β*	*p-value*	*β*	*p-value*	*β*	*p-value*	*β*	*p-value*
Abdominal fat distribution assessed by ultrasound.										
Total abdominal fat, *cm*	0.532	**<0.001**	0.676	**<0.001**	–	–	-0.572	**<0.001**	-0.374	**0.001**
Subcutaneous adipose tissue, *cm*	0.280	*0.063*	0.311	**0.039**	–	**-**	-0.402	**0.005**	-0.356	**0.015**
Visceral adipose tissue, *cm*	0.506	**<0.001**	0.664	**<0.001**	–	**-**	-0.498	**<0.001**	-0.283	*0.053*
VAT/SAT ratio	–	–	–	–	–	**-**	–	–	–	–
Abdominal fat distribution assessed by routinely applicable anthropometric parameters.										
Body mass index, *kg/m^2^*	0.700	**<0.001**	0.868	**<0.001**	–	–	-0.672	**<0.001**	-0.423	**0.002**
Waist circumference, *cm*	0.595	**<0.001**	0.714	**<0.001**	–	**-**	-0.598	**<0.001**	-0.510	**<0.001**
Weight-adjusted-Waist Index	0.403	**0.008**	0.478	**0.002**	–	–	-0.396	**0.006**	-0.447	**0.002**
Waist-to-height ratio	0.609	**<0.001**	0.757	**<0.001**	–	–	-0.586	**<0.001**	-0.496	**<0.001**
Conicity Index	0.394	**0.008**	0.444	**0.003**	–	–	-0.036	0.790	-0.410	**0.003**

SMI, Skeletal Muscle Mass Index; ASMI, Appendicular Skeletal Muscle Mass Index, HGS, Handgrip Strength; BW, Body Weight; GS, Gait Speed; VAT, Visceral Adipose Tissue; SAT, Subcutaneous Adipose Tissue. Linear regression was adjusted for age and total physical activity (METs/week), a p-value <0.05 is statistically significant. Significant results are expressed in **bold**, borderline results are expressed in *italic*.

After linear regression, statistically significant positive associations were observed for total abdominal fat and SMI (*β* = 0.532, p <0.001), ASMI (*β* = 0.676, p <0.001) and an inverse association with HGS/BW (*β* = -0.572, p <0.001) and GS (*β* = -0.374, p=0.001).

Regarding SAT, after linear regression, statistically significant positive associations were observed for ASMI (*β* = 0.311, p=0.039) and a significant inverse association with HGS/BW (*β* = -0.402, p=0.005) and GS (*β* = -0.356, p=0.015).

Lastly, regarding VAT, after linear regression, statistically significant positive associations were observed for SMI (*β* = 0.506, p <0.001), ASMI (*β* = 0.664, p <0.001), and an inverse significant association with HGS/BW (*β* = -0.498, p <0.001).

### 3.5 Association between abdominal fat distribution assessed by routinely applicable anthropometric parameters and sarcopenia parameters in all population

Spearman correlation analysis in all population are presented in **Table 1** and linear regression analysis in all population are presented in **Table 3**.

After linear regression, statistically significant positive associations were observed for BMI and SMI (*β* = 0.590, p <0.001), ASMI (*β* = 0.628, p <0.001) and an inverse association with HGS/BW (*β* = -0.478, p <0.001) and GS (*β* = -0.380, p=0.001).

Regarding WC, after linear regression, statistically significant positive associations were observed for SMI (*β* = 0.524, p <0.001), ASMI (*β* = 0.544, p < 0.001) and a significant inverse association for GS (*β* = -0.483, p < 0.001).

Regarding WWI, after linear regression, statistically significant positive association were observed for ASMI (*β* = 0.276, p=0.003) and significant inverse associations for HGS/BW (*β* = -0.304, p=0.002) and GS (*β* = -0.441, p < 0.001).

Regarding WHtR, after linear regression, statistically significant positive association was observed for ASMI (*β* = 0.509, p <0.001) and significant inverse associations for HGS/BW (*β* = -0.444, p < 0.001) and GS (*β* = -0.471, p < 0.001).

Lastly, regarding CI, after linear regression, statistically significant positive association were observed for SMI (*β* = 0.292, p=0.004), ASMI (*β* = 0.279, p=0.002) and a significant inverse association with GS (*β* = -0.441, p < 0.001).

### 3.6 Association between abdominal fat distribution assessed by routinely applicable anthropometric parameters and sarcopenia parameters in men

Spearman correlation analysis in men are presented in **Table 2** and linear regression analysis in men are presented in **Table 4**.

After linear regression, statistically significant positive associations were observed for BMI and SMI (*β* = 0.846, p <0.001), ASMI (*β* = 0.901, p <0.001) and an inverse association with HGS/BW (*β* = -0.533, p=0.009).

Regarding WC, after linear regression, statistically significant positive associations were observed for SMI (*β* = 0.769, p <0.001), ASMI (*β* = 0.825, p < 0.001) and a significant inverse association with HGS/BW (*β* = -0.634, p=0.003).

Regarding WHtR, after linear regression, statistically significant positive associations were observed for SMI (*β* = 0.864, p <0.001), ASMI (*β* = 0.851, p <0.001) and a significant inverse association with HGS/BW (*β* = -0.557, p=0.019).

Regarding, CI, after linear regression, a significant inverse association with HGS/BW (*β* = -0.596, p=0.022) was observed.

Lastly, regarding WWI, after linear regression, no statistically significant associations were observed.

### 3.7 Association between abdominal fat distribution assessed by routinely applicable anthropometric parameters and sarcopenia parameters in women

Spearman correlation analysis in women are presented in **Table 3** and linear regression analysis in women are presented in **Table 5**.

After linear regression, statistically significant positive associations were observed for BMI and SMI (*β* = 0.700, p <0.001), ASMI (*β* = 0.868, p <0.001) and inverse associations with HGS/BW (*β* = -0.672, p <0.001) and GS (*β* = -0.426, p=0.002).

Regarding WC, after linear regression, statistically significant positive association were observed for SMI (*β* = 0.595, p <0.001), ASMI (*β* = 0.714, p <0.001) and significant inverse association with HGS/BW (*β* = -0.598, p <0.001) and GS (*β* = -0.510, p <0.001).

Regarding WWI, after linear regression, statistically significant positive association were observed for SMI (*β* = 0.403, p=0.008), ASMI (*β* = 0.478, p=0.002) and significant inverse associations with HGS/BW (*β* = -0.396, p=0.006) and GS (*β* = -0.447, p=0.002).

Regarding WHtR, after linear regression, statistically significant positive association were observed for SMI (*β* = 0.609, p <0.001), ASMI (*β* = 0.757, p <0.001) and significant inverse associations with HGS/BW (*β* = -0.586, p <0.001) and GS (*β* = -0.496, p <0.001).

Lastly, regarding, CI, after linear regression, statistically significant positive associations were observed for SMI (*β* = 0.94, p=0.008), ASMI (*β* = 0.444, p=0.003), and an inverse moderate correlation with GS (*β* = -0.410, p=0.003).

## 4 Discussion

The present cross-sectional study provides novel insights into the relationship between abdominal fat distribution, assessed by ultrasound and routinely applicable anthropometric parameters, and sarcopenia-related parameters in older adults. The present study results show that different parameters of abdominal adiposity are differentially associated with sarcopenic parameters such as muscle mass, strength, and physical performance, with notable sex-specific patterns.

In all population, increased abdominal fat, total abdominal fat, SAT, and VAT, were positively associated with muscle mass indices (SMI and ASMI) while total abdominal fat and SAT being inversely associated with physical performance (GS) and relative muscle strength (HGS/BW).

These findings support the hypothesis that increased adiposity may coexist with preserved or even elevated muscle mass, while simultaneously contributing to functional decline via loss of muscle strength and physical performance. Although 11.3% of the participants (8/72) present low GS and increased WC, they are not classified as sarcopenic according to any consensus. This suggests a dissociation between muscle quantity, quality, and physical performance, differing from classical sarcopenic obesity, where both increased adiposity and reduced muscle mass are typically present (13). Instead, it aligns with evidence describing age-related adipose redistribution, in which fat preferentially accumulates in the abdominal region while muscle mass may remain within normal ranges, but declining functional capacity (31). This phenotype appears particularly relevant in individuals aged 65–75 years, a transitional stage in which many older adults do not yet meet diagnostic criteria for sarcopenia although some parameters are already diminished, but already exhibit measurable changes in body composition and function. This period represents a critical phase in the trajectory toward frailty, during which early functional decline becomes evident (32).

With advanced age, adipose tissue inflammation contributes to the redistribution of body fat toward the intra-abdominal (visceral) compartment and to increased lipid infiltration within skeletal muscle, thereby resulting in a decline in overall muscular strength and physical function (31). These redistribution changes can occur independently of a formal sarcopenia diagnosis and are often referred to as body composition remodelling with ageing (33).

**Figure 2:**
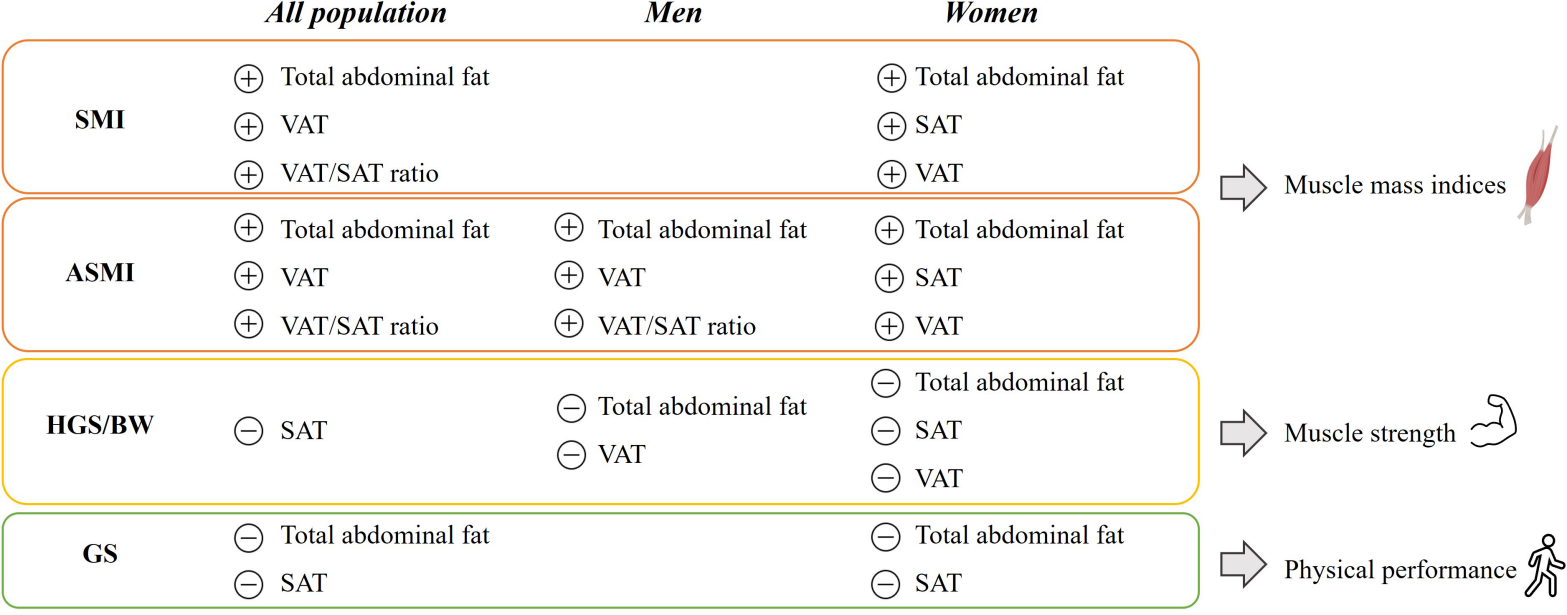
Linear regression analysis between ultrasound-assessed abdominal fat distribution and sarcopenia parameters. SMI, Skeletal Muscle Index; ASMI; Appendicular Skeletal Mass Index; HGS, Handgrip Strength; HGS/BW; Body Weight adjusted Handgrip strength; GS, Gait Speed; SAT, Subcutaneous Adipose Tissue; VAT, Visceral Adipose Tissue. Only significant linear regression results are presented, a p < 0.05 is considered significant. ⊕Positive linear regression; ⊖ negative linear regression.

In the present study, VAT showed stronger positive associations with sarcopenia-related parameters of muscle mass, whereas SAT showed more consistent inverse associations with muscle strength and physical performance in all population. Although SAT showed weaker and less consistent associations with muscle mass indices (SMI and ASMI) compared to VAT, it was negatively associated with relative muscle strength (HGS/BW) and physical performance (GS), while these associations were not seen for VAT in all population (**Figure 2**).

GS is considered an early marker of frailty, being a strong predictor of disability, dependence, and adverse health outcomes in older adults (34, 35). These findings are consistent with the physiological differences and functional properties of VAT and SAT, and how they respond differently to ageing processes, supporting the idea that adipose compartments do not behave uniformly (36, 37). SAT undergoes specific ageing-related changes in structure and cellular function, with reduced adipogenesis, chronic inflammatory signalling, and diminished lipid-buffering capacity, therefore, impairing its ability to expand and store lipids efficiently due to reduced adipocyte function and increased fibrosis (38, 39). Consequently, as SAT losses plasticity and reduces its buffering capacity, excess lipids are redirected to visceral depots, where VAT expands and exhibits enhanced immune cell infiltration and cytokine secretion, contributing to inflammation and metabolic dysfunction (38, 40). Additionally, the reduced lipid-buffering capacity of SAT promotes the ectopic deposition of lipids within skeletal muscle, leading to myosteatosis, mitochondrial dysfunction, and impaired insulin signalling, all of which compromise muscle quality and regenerative capacity (31, 38). These alterations translate into reduced muscle strength and contractile efficiency, contributing to declines in physical performance, including slower GS and increased frailty risk in older adults (31).Thus, SAT dysfunction not only drives VAT expansion and systemic metabolic impairment but also plays a central role in the development of sarcopenia and mobility limitation.

Sex-specific analyses revealed important differences in the relationship between abdominal fat distribution and sarcopenia-related parameters (**Figure 2**).

In men, VAT and total abdominal fat were more strongly inversely associated with relative muscle strength (HGS/BW), whereas associations with GS were less consistent.

In women, associations were more uniform and extended across both SAT and VAT. A consistent inverse relationship was seen between, strength (HGS/BW) and both VAT and SAT, whereas an inverse relationship was only seen between SAT and physical performance (GS).

These findings may reflect sex-specific differences in fat distribution, hormonal status, and muscle physiology, particularly in older age, and suggest that the mechanisms linking adiposity and sarcopenia may differ between men and women (41, 42). Therefore, in early older age, fat redistribution rather than sarcopenic obesity may be a key determinant of functional decline. Also, in the present study, no participants met the diagnostic criteria for confirmed sarcopenic obesity, despite 65.7% exhibiting elevated WC and 23.6% being classified as obese. It should be noted that that the diagnostic criterion used to define obesity, whether BMI or WC, can substantially influence classification outcomes, particularly in older adults, for whom BMI thresholds differ from those used in the general adult population.

**Figure 3:**
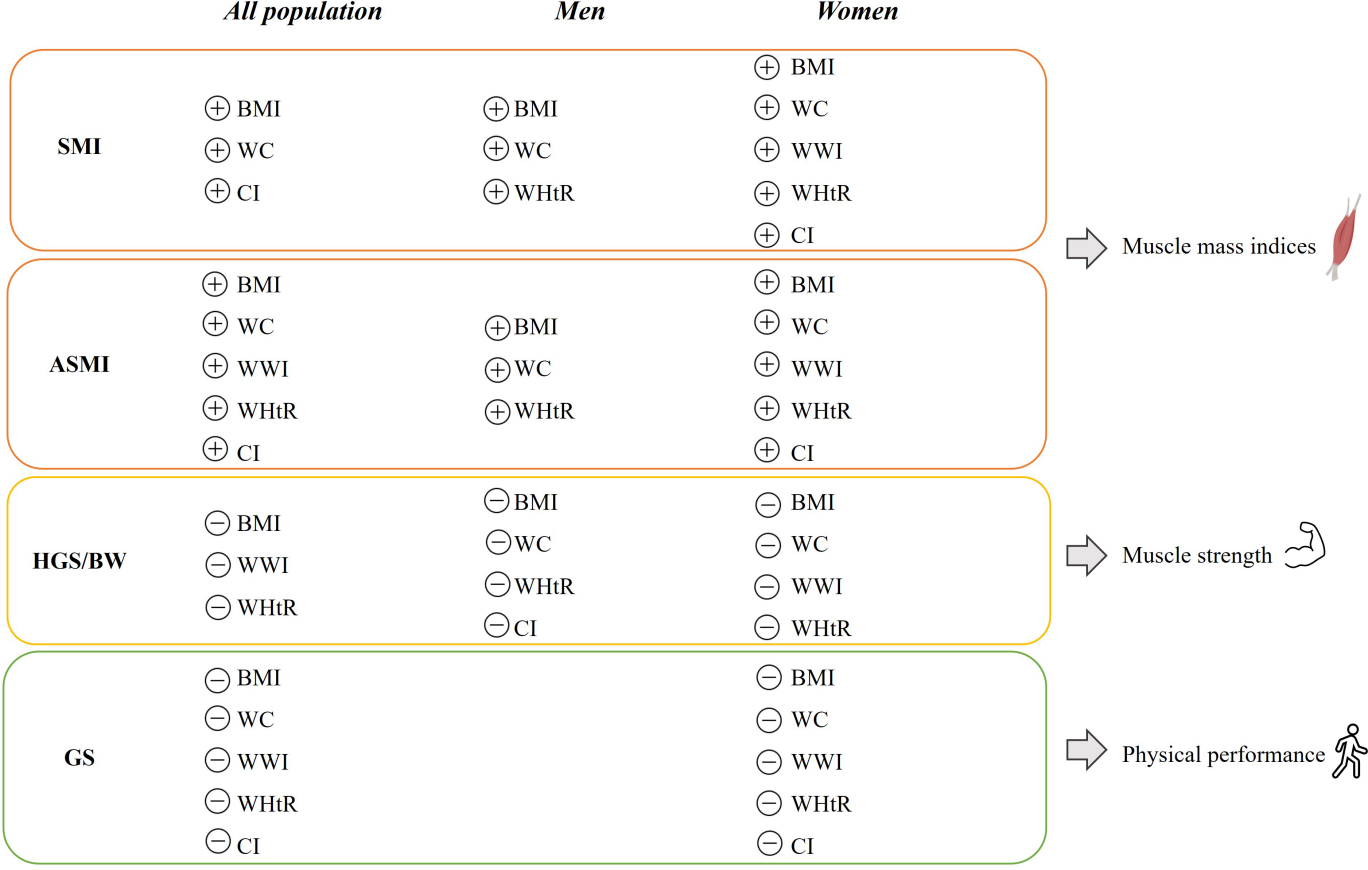
Linear regression analysis between routinely applicable anthropometric parameters and sarcopenia parameters. SMI, Skeletal Muscle Index; ASMI; Appendicular Skeletal Mass Index; HGS, Handgrip Strength; HGS/BW; Body Weight adjusted Handgrip strength; GS, Gait Speed; BMI, Body Mass Index; WC, Waist Circumference, WWI; Weight-adjusted Waist; WHtR, Waist-to-Height Ratio; CI, Conicity Index. Only significant linear regression results are presented, a p < 0.05 is considered significant. ⊕Positive linear regression; ⊖ negative linear regression.

Anthropometric measures such as BMI, WC, WHtR, WWI, and CI also showed significant associations with sarcopenia-related parameters. Notably, these outcomes were positively associated with muscle mass (SMI and ASMI) and inversely associated with relative muscle strength (HGS/BW) and physical performance (GS), mirroring the patterns observed with ultrasound-measurements of abdominal fat distribution (**Figure 3**).

Among these, WC and WHtR showed particularly strong and consistent associations, these results being consistent with previous research, reinforcing their utility as practical, low-cost tools for identifying individuals at risk of sarcopenia or sarcopenic obesity in clinical and community settings (43). The results of the present study are consistent with a study conducted by Baş et al., which reported that SAT measured by ultrasound is associated with anthropometric indices such as BMI and WC, and an inverse association was observed between SAT and HGS, but only in participants with obesity (44). In contrast, in the present study, SAT is significantly associated not only with muscle mass but also with muscle strength (HGS/BW) and physical performance (GS), in all population and in women. These results suggest that, unlike previously reported, SAT may have a more relevant functional impact in the development of sarcopenia due to the loss of plasticity and increased fibrosis, therefore reducing buffering capacity of SAT and consequently redirecting excess lipids to VAT and within skeletal muscle (ectopic fat), contributing to declines in physical function, muscle strength and contractile efficiency (31, 38). Moreover, SAT in obesity secretes factors like resistin that disrupt muscle cell differentiation, reduces fibre growth, and promotes inflammatory pathways (45, 46). While younger muscle can compensate, aging muscle is more vulnerable due to chronic low-grade inflammation, reduced stem cell function, anabolic resistance, and impaired repair capacity (45, 46). As a result, SAT’s large volume and altered biology make it a meaningful contributor to systemic inflammation and age-related muscle loss (sarcopenia), highlighting that fat dysfunction, not just fat location, is key to metabolic and musculoskeletal health (45, 46), supporting the use of abdominal ultrasound in the assessment of sarcopenia.

Our results are consistent with previous literature, suggesting that increased adiposity, especially central obesity, is linked to reduced muscle function and physical performance (47). Furthermore, our findings may reflect a specific abdominal adiposity phenotype in older adults, characterized by increased abdominal fat accumulation relatively preserved skeletal muscle mass, and low physical performance (8/72; 14.3% women). This interpretation is supported by evidence indicating that age-related changes in body composition are compartment-specific, with increases in adiposity occurring earlier, while significant declines in lean mass may appear later in the ageing trajectory (48).

Notably, functional decline in older adults is not determined solely by muscle mass. A systematic review demonstrated that adiposity and low muscle strength are strongly associated with functional decline, whereas muscle mass alone shows weaker or inconsistent associations with physical performance and disability outcomes (49). This suggests that individuals may experience impaired physical performance despite relatively preserved muscle mass, particularly during the early stages of ageing. Additionally, the observed positive associations between adiposity and muscle mass indices further emphasize the complexity of the relationship between fat and muscle in ageing populations.

This study has several notable strengths. First, it provides a comprehensive assessment of abdominal adiposity using both imaging techniques such as ultrasound and widely accessible anthropometric measures, allowing for direct comparison of their associations with sarcopenia-related outcomes.

Second, the simultaneous evaluation of muscle mass, strength, and physical performance provides a multidimensional assessment of sarcopenia in line with current consensus definitions.

Third, the inclusion of sex-stratified analyses enables the identification of important differences between men and women, which enhances the clinical relevance of the findings.

Nonetheless, several limitations should be acknowledged. The cross-sectional design of the study precludes any inference of causality, and longitudinal studies are required to determine the temporal relationship between abdominal fat distribution and sarcopenia progression. Furthermore, the relatively small sample size, 72 participants and a predominantly female population (49/72, 68.1%) from Spain, may limit statistical power and the generalizability of the findings, particularly in sex-stratified analyses. A limitation of the statistical approach is that normality was assessed using the Kolmogorov–Smirnov test, whereas the Shapiro–Wilk test may have been more appropriate for smaller subgroups, particularly the male subgroup (n = 23). Another limitation of this study is that regression analyses were performed only for statistically significant correlations, which may introduce selection bias. In addition, no correction for multiple comparisons was applied in the exploratory correlation analyses, which may increase the risk of type I error. Additionally, regarding the generalisability of our findings, it is important to acknowledge that this study was conducted in a single-centre Spanish population from the Camp de Tarragona region, which may influence abdominal fat distribution due to specific ethnic, lifestyle, and dietary characteristics. The population of Mediterranean regions typically shows distinctive lifestyle and dietary patterns, such as moderate physical activity and high adherence to the Mediterranean diet, which have been associated with more favourable fat distribution profiles in previous studies (50).

Also, although ultrasound provides a non-invasive and practical method for assessing fat distribution, it is operator-dependent and may introduce measurement variability. Additionally, in this study, US- measurement of VAT assessed a combined anterior intra-abdominal fat compartment and did not allow reliable differentiation between preperitoneal and omental fat. Given the potentially distinct metabolic and physiological roles of these compartments, future studies should evaluate them separately. Furthermore, in this study, the measurement of lipid infiltration by ultrasound within skeletal muscle was not assessed, but future studies should evaluate lipid infiltration in order to determine changes in fat distribution with early ageing.

Interestingly, while simple anthropometric indices such as WC and WHtR showed strong and consistent associations with sarcopenia-related parameters, they do not differentiate between SAT and VAT, whereas ultrasound-derived measures provide detailed insights into fat distribution and how each fat compartment affects distinct sarcopenia-related parameters, supporting its use as a practical screening tool in clinical practice. These relationships were evident across the whole population and differed by sex, with women showing more consistent associations across fat compartments with relative muscle strength and physical function, while men showed stronger links between adiposity and relative muscle strength. Future longitudinal research is warranted to clarify the causal pathways and to explore potential interventions targeting both adiposity and muscle function in older adults.

## 5 Conclusion

In conclusion, the present cross-sectional study shows that abdominal fat distribution is differentially associated with sarcopenia-related parameters.

Ultrasound-assessed abdominal fat distribution showed sex-specific associations with sarcopenia parameters. VAT showed the strongest positive association with muscle mass indices, while SAT was the only depot associated with lower relative muscle strength and physical performance in all population. Sex-specific results showed that, in men and in women, VAT was positively associated with muscle mass and negatively associated with relative muscle strength. Whereas in women, SAT was the only fat depot negatively associated with physical performance.

These results suggest that abdominal fat distribution may influence physical performance and relative muscle strength in ageing populations, highlighting the importance of evaluating abdominal fat distribution, in addition to total adiposity, when assessing sarcopenia parameters.

## Data Availability

The raw data supporting the conclusions of this article will be made available by the authors, without undue reservation.
