# Supplementary material for: Ultrasound-assessed abdominal fat distribution and its relation to sarcopenia parameters in community-dwelling young older adults: a cross-sectional study
Source: Front Endocrinol (Lausanne). 2026 Jun 19;17:1888492. doi: 10.3389/fendo.2026.1888492 (PMC13327918; doi:10.3389/fendo.2026.1888492)
Supplement: Supplementary file 1 [file SupplementaryFile1.docx]

**Supplementary File 1.**

- 1. *Association between abdominal fat distribution assessed by ultrasound and sarcopenia parameters in all population*

Spearman correlation analysis in all population are presented in **Supplementary Table 1**.

Total abdominal fat presented a moderate positive correlation with SMI (*r*=0.588, p <0.001), a high correlation with ASMI (*r*=0.636, p <0.001), a borderline weak positive correlation with HGS (*r*=0.228, p=0.060), and an inverse weak correlation with GS (*r*= -0.266, p=0.027).

SAT presented an inverse weak correlation with HGS (*r*= -0.251, p=0.038), and GS (*r*= -0.331, p=0.006), and an inverse moderate correlation with HGS/BW (*r*= -0.433, p <0.001).

VAT presented a high positive correlation with SMI (*r*=0.610, p <0.001), ASMI (*r*=0.685, p <0.001), and a moderate positive correlation with HGS (*r*=0.316, p=0.008).

VAT/SAT ratio presented a moderate positive correlation with SMI (*r*=0.432, p <0.001), ASMI (*r*=0.511, p <0.001), and HGS (*r*=0.353, p=0.003).

- 1. *Association between abdominal fat distribution assessed by ultrasound and sarcopenia parameters in men*

Spearman correlation analysis in men are presented in **Supplementary Table 2**.

Total abdominal fat presented a moderate positive correlation with ASMI (*r*=0.486, p=0.022), an inverse moderate correlation with HGS/BW (*r*= -0.557, p=0.007), and an inverse weak correlation for GS (*r*= -0.244, p=0.007).

VAT presented a moderate positive correlation with SMI (*r*=0.439, p=0.041), ASMI (*r*=0.512, p=0.015), and an inverse moderate correlation with HGS/BW (*r*= -0.544, p=0.009).

VAT/SAT ratio presented a borderline moderate positive correlation with ASMI (*r*=0.420, p=0.052).

No significant correlations were observed for SAT.

- 1. *Association between abdominal fat distribution assessed by ultrasound and sarcopenia parameters in women*

Spearman correlation analysis in women are presented in **Supplementary Table 3**.

Total abdominal fat presented a moderate positive correlation with SMI (*r*=0.581, p <0.001), a high positive correlation with ASM (*r*=0.636, p <0.001), and an inverse moderate correlation with HGS/BW (*r*= -0-405, p=0.005) and GS (*r*= -0.366, p=0.011).

SAT presented a moderate positive correlation with SMI (*r*=0.344, p=0.018) and ASMI (*r*=0.350, p=0.016), and an inverse moderate correlation with HGS/BW (*r*=-0.343, p=0.018) and GS (*r*= -0.363, p=0.012).

VAT presented a moderate positive correlation with SMI (*r*=0.525, p <0.001), ASMI (*r*=0.603, p <0.001), an inverse moderate correlation with HGS/BW (*r*= -0.343, p=0.018), and a borderline inverse moderate correlation with GS (*r*= -0.286, p=0.052)

No significant correlations were observed for VAT/SAT ratio.

- 1. *Association between abdominal fat distribution assessed by routinely applicable anthropometric parameters and sarcopenia parameters in all population*

Spearman correlation analysis in all population are presented in **Supplementary Table 1**.

BMI presented a high positive correlation with SMI (*r*=0.701, p<0.001), ASMI (*r*=0.710, p <0.001), and an inverse moderate correlation with HGS/BW (*r*= -0.431, p <0.001) and GS (*r*= -0.365, p=0.002).

WC presented a high positive correlation with SMI (*r*=0.715, p <0.001), ASMI (*r*=0.753, p <0.001), a moderate positive correlation with HGS (*r*=0.316, p=0.008) and an inverse moderate correlation with GS (*r*= -0.316, p=0.008).

WWI presented a borderline weak positive correlation with ASMI (*r*=0.229, p=0.056) and an inverse moderate correlation with HGS/BW (*r*= -0.444, p <0.001) and GS (*r*= -0.470, p <0.001).

WHtR presented a moderate positive correlation with SMI (*r*=0.483, p <0.001), ASMI (*r*=0.508, p <0.001), and an inverse moderate correlation with HGS/BW (*r*= -0.527, p <0.001) and GS (*r*= -0.466, p= < 0.001).

CI presented a moderate positive correlation with SMI (*r*=0.411, p <0.001), ASMI (*r*=0.454, p <0.001), and an inverse moderate correlation with GS (*r*= -0.368, p=0.002).

- 1. *Association between abdominal fat distribution assessed by routinely applicable anthropometric parameters and sarcopenia parameters in men*

Spearman correlation analysis in men are presented in **Supplementary Table 2**.

BMI presented a high positive correlation with SMI (*r*=0.843, p <0.001), ASMI (*r*=0.944, p <0.001), and an inverse high correlation with HGS/BW (*r*= -0.621, p=0.002).

WC presented high positive correlations with SMI (*r*=0.658, p=0.001), ASMI (*r*=0.836, p <0.001), and an inverse high correlation with HGS/BW (*r*= -0.745, p <0.001).

WWI presented an inverse moderate correlation with HGS/BW (*r*= -0.517, p=0.014).

WHtR presented a moderate positive correlation with SMI (*r*=0.583, p=0.004), a high positive correlation with ASMI (*r*=0.800, p <0.001), and an inverse high correlation with HGS/BW (*r*= -0.686, p <0.001).

CI presented an inverse moderate correlation with HGS (*r*= -0.466, p=0.029), and an inverse high correlation with HGS/BW (*r*= -0.652, p=0.001).

- 1. *Association between abdominal fat distribution assessed by routinely applicable anthropometric parameters and sarcopenia parameters in women*

Spearman correlation analysis in women are presented in **Supplementary Table 3**.

BMI presented a moderate positive correlation with SMI (*r*=0.527, p <0.001), a high positive correlation with ASMI (*r*=0.821, p <0.001), and an inverse high correlation with HGS/BW (*r*= -0.629, p <0.001), and an inverse moderate correlation with GS (*r*= -0.447, p=0.001).

WC presented a moderate positive correlation with SMI (*r*=0.591, p <0.001), a high positive correlation with ASMI (*r*=0.661, p <0.0012), and inverse moderate correlations with HGS/BW (*r*= -0.541, p <0.001) and GS (*r*= -0.516, p <0.001).

WWI presented moderate positive correlations with SMI (*r*=0.350, p=0.015), ASMI (*r*=0.401, p=0.005), and inverse moderate correlations with HGS/BW (*r*= -0.426, p=0.003) and GS (*r*= -0.491, p <0.001).

WHtR presented a moderate positive correlation with SMI (*r*=0.567, p <0.001), a high positive correlation with ASMI (*r*=0.680, p <0.001), and inverse moderate correlations with HGS/BW (*r*= -0.590, p <0.001) and GS (*r*= -0.527, p <0.001).

CI presented moderate positive correlations with SMI (*r*=0.366, p=0.011), ASMI (*r*=0.399, p=0.005), and inverse moderate correlation with HGS/BW (*r*= -0.421, p=0.003) and GS (*r*= -0.507, p <0.001).
