# Supplementary material for: Ultrasound-assessed abdominal fat distribution and its relation to sarcopenia parameters in community-dwelling young older adults: a cross-sectional study
Source: Front Endocrinol (Lausanne). 2026 Jun 19;17:1888492. doi: 10.3389/fendo.2026.1888492 (PMC13327918; doi:10.3389/fendo.2026.1888492)
Supplement: Supplementary file 2 [file SupplementaryFile2.docx]

**Supplementary Table 1. Spearman correlations between abdominal fat distribution and sarcopenia parameters in all population.**

|  | **Sarcopenia parameters** | | | | | | | | | |
| --- | --- | --- | --- | --- | --- | --- | --- | --- | --- | --- |
|  | **SMI**, *kg/m^2^* | | **ASMI**, *kg/m^2^* | | **HGS**, *kg* | | **HGS/BW** | | **GS**, *m/s* | |
|  | *r* | *p-value* | *r* | *p-value* | *r* | *p-value* | *r* | *p-value* | *r* | *p-value* |
| **Abdominal fat distribution assessed by ultrasound** | | | | | | | | | | |
| Total abdominal fat, *cm* | 0.588 | **<0.001** | 0.636 | **<0.001** | 0.228 | *0.060* | -0.212 | 0.081 | -0.266 | **0.027** |
| Subcutaneous adipose tissue, *cm* | 0.014 | 0.908 | -0.036 | 0.771 | -0.251 | **0.038** | -0.433 | **<0.001** | -0.331 | **0.006** |
| Visceral adipose tissue, *cm* | 0.610 | **<0.001** | 0.685 | **<0.001** | 0.322 | **0.007** | -0.085 | 0.489 | -0.183 | 0.131 |
| VAT/SAT ratio | 0.432 | **<0.001** | 0.511 | **<0.001** | 0.353 | **0.003** | 0.221 | 0.069 | 0.054 | 0.661 |
| **Abdominal fat distribution assessed by routinely applicable anthropometric parameters** | | | | | | | | | | |
| Body mass index, *kg/m^2^* | 0.701 | **<0.001** | 0.710 | **<0.001** | 0.075 | 0.532 | -0.431 | **<0.001** | -0.365 | **0.002** |
| Waist circumference, *cm* | 0.715 | **<0.001** | 0.753 | **<0.001** | 0.316 | **0.008** | -0.201 | 0.096 | -0.316 | **0.008** |
| Weight-adjusted-Waist Index | 0.211 | 0.080 | 0.229 | *0.056* | -0.209 | 0.082 | -0.444 | **<0.001** | -0.470 | **<0.001** |
| Waist-to-height ratio | 0.483 | **<0.001** | 0.508 | **<0.001** | -0.104 | 0.391 | -0.527 | **<0.001** | -0.466 | **<0.001** |
| Conicity Index | 0.411 | **<0.001** | 0.454 | **<0.001** | 0.128 | 0.290 | -0.213 | 0.077 | -0.368 | **0.002** |

SMI, Skeletal Muscle Mass Index; ASMI, Appendicular Skeletal Muscle Mass Index, HGS, Handgrip Strength; BW, Body Weight; GS, Gait Speed; VAT, Visceral Adipose Tissue; SAT, Subcutaneous Adipose Tissue. A p-value <0.05 is statistically significant. Significant results are expressed in **bold**, borderline results are expressed in *italic*.

**Supplementary Table 2. Spearman correlations between abdominal fat distribution and sarcopenia parameters in men.**

|  | **Sarcopenia parameters** | | | | | | | | | |
| --- | --- | --- | --- | --- | --- | --- | --- | --- | --- | --- |
|  | **SMI**, *kg/m^2^* | | **ASMI**, *kg/m^2^* | | **HGS**, *kg* | | **HGS/BW** | | **GS**, *m/s* | |
|  | *r* | *p-value* | *r* | *p-value* | *r* | *p-value* | *r* | *p-value* | *r* | *p-value* |
| **Abdominal fat distribution assessed by ultrasound** | | | | | | | | | | |
| Total abdominal fat, *cm* | 0.310 | 0.160 | 0.486 | **0.022** | -0.164 | 0.465 | -0.557 | **0.007** | -0.244 | **0.007** |
| Subcutaneous adipose tissue, *cm* | 0.063 | 0.782 | -0.019 | 0.934 | -0.023 | 0.919 | -0.095 | 0.673 | -0.035 | 0.877 |
| Visceral adipose tissue, *cm* | 0.439 | **0.041** | 0.512 | **0.015** | -0.132 | 0.558 | -0.544 | **0.009** | -0.141 | 0.531 |
| VAT/SAT ratio | 0.244 | 0.273 | 0.420 | *0.052* | 0.013 | 0.954 | -0.296 | 0.180 | -0.084 | 0.710 |
| **Abdominal fat distribution assessed by routinely applicable anthropometric parameters** | | | | | | | | | | |
| Body mass index, *kg/m^2^* | 0.843 | **<0.001** | 0.944 | **<0.001** | 0.062 | 0.779 | -0.621 | **0.002** | -0.233 | 0.297 |
| Waist circumference, *cm* | 0.658 | **0.001** | 0.836 | **<0.001** | -0.089 | 0.695 | -0.745 | **<0.001** | -0.161 | 0.487 |
| Weight-adjusted-Waist Index | 0.125 | 0.580 | 0.285 | 0.198 | -0.319 | 0.072 | -0.517 | **0.014** | -0.309 | 0.174 |
| Waist-to-height ratio | 0.583 | **0.004** | 0.800 | **<0.001** | -0.149 | 0.508 | -0.686 | **<0.001** | -0.214 | 0.352 |
| Conicity Index | 0.169 | 0.453 | 0.340 | 0.121 | -0.466 | **0.029** | -0.652 | **0.001** | -0.263 | 0.248 |

SMI, Skeletal Muscle Mass Index; ASMI, Appendicular Skeletal Muscle Mass Index, HGS, Handgrip Strength; BW, Body Weight; GS, Gait Speed; VAT, Visceral Adipose Tissue; SAT, Subcutaneous Adipose Tissue. A p-value <0.05 is statistically significant. Significant results are expressed in **bold**, borderline results are expressed in *italic*.

**Supplementary Table 3. Spearman correlations between abdominal fat distribution and sarcopenia parameters in women.**

|  | **Sarcopenia parameters** | | | | | | | | | |
| --- | --- | --- | --- | --- | --- | --- | --- | --- | --- | --- |
|  | **SMI**, *kg/m^2^* | | **ASMI**, *kg/m^2^* | | **HGS**, *kg* | | **HGS/BW** | | **GS**, *m/s* | |
|  | *r* | *p-value* | *r* | *p-value* | *r* | *p-value* | *r* | *p-value* | *r* | *p-value* |
| **Abdominal fat distribution assessed by ultrasound** | | | | | | | | | | |
| Total abdominal fat, *cm* | 0.581 | **<0.001** | 0.636 | **<0.001** | 0.066 | 0.660 | -0.405 | **0.005** | -0.366 | **0.011** |
| Subcutaneous adipose tissue, *cm* | 0.344 | **0.018** | 0.350 | **0.016** | 0.052 | 0.727 | -0.322 | **0.027** | -0.363 | **0.012** |
| Visceral adipose tissue, *cm* | 0.525 | **<0.001** | 0.603 | **<0.001** | 0.056 | 0.711 | -0.343 | **0.018** | -0.286 | *0.052* |
| VAT/SAT ratio | 0.182 | 0.220 | 0.207 | 0.163 | -0.080 | 0.595 | -0.033 | 0.825 | 0.038 | 0.798 |
| **Abdominal fat distribution assessed by routinely applicable anthropometric parameters** | | | | | | | | | | |
| Body mass index, *kg/m^2^* | 0.527 | **<0.001** | 0.821 | **<0.001** | -0.051 | 0.729 | -0.629 | **<0.001** | -0.447 | **0.001** |
| Waist circumference, *cm* | 0.591 | **<0.001** | 0.661 | **<0.001** | 0.016 | 0.911 | -0.541 | **<0.001** | -0.516 | **<0.001** |
| Weight-adjusted-Waist Index | 0.350 | **0.015** | 0.401 | **0.005** | -0.217 | 0.139 | -0.426 | **0.003** | -0.491 | **<0.001** |
| Waist-to-height ratio | 0.567 | **<0.001** | 0.670 | **<0.001** | -0.159 | 0.280 | -0.590 | **<0.001** | -0.527 | **<0.001** |
| Conicity Index | 0.366 | **0.011** | 0.399 | **0.005** | -0.100 | 0.498 | -0.421 | **0.003** | -0.507 | **<0.001** |

SMI, Skeletal Muscle Mass Index; ASMI, Appendicular Skeletal Muscle Mass Index, HGS, Handgrip Strength; BW, Body Weight; GS, Gait Speed; VAT, Visceral Adipose Tissue; SAT, Subcutaneous Adipose Tissue. A p-value <0.05 is statistically significant. Significant results are expressed in **bold**, borderline results are expressed in *italic*.
